# Correlation Between Urinary Osteopontin Concentration and the Mineral Content and Composition of Kidney Stones

**DOI:** 10.3390/jcm14176247

**Published:** 2025-09-04

**Authors:** Maciej Jaromin, Piotr Kutwin, Tomasz Konecki, Hanna Jerczyńska, Piotr Wysocki, Magdalena Gajek, Waldemar Maniukiewicz, Małgorzata Iwona Szynkowska-Józwik, Dariusz Moczulski

**Affiliations:** 1 1st Department of Urology, Medical University of Lodz, 90-549 Lodz, Poland; maciej.jaromin@stud.umed.lodz.pl (M.J.); tomasz.konecki@umed.lodz.pl (T.K.); 2 Research Laboratory CoreLab, Medical University of Lodz, 92-215 Lodz, Poland; hanna.jerczynska@umed.lodz.pl; 3 Faculty of Chemistry, Institute of General and Ecological Chemistry, Lodz University of Technology, 90-534 Lodz, Poland; piotr.wysocki@p.lodz.pl (P.W.); magdalena.gajek@p.lodz.pl (M.G.); waldemar.maniukiewicz@p.lodz.pl (W.M.); malgorzata.szynkowska@p.lodz.pl (M.I.S.-J.); 4 Department of Internal Medicine and Nephrodiabetology, University Clinical Hospital No. 2 of the Medical University of Lodz, 90-549 Lodz, Poland; dariusz.moczulski@umed.lodz.pl

**Keywords:** urolithiasis, kidney stone disease, osteopontin, calcium oxlalate, calcium phosphate

## Abstract

**Background and Objective**: Information about the type of kidney stones is important for informed therapeutic decisions and the prevention of urolithiasis. Urinary stones are heterogeneous, and their elemental composition and crystal structure vary between patients. The formation of urinary stone deposits depends, among other things, on physiological conditions, the concentration of promoters and inhibitors of crystallization, and proteins found in the urine. The aim of this study was to determine differences in urine osteopontin (OPN) levels between groups of different stone-formers. **Methods**: Urinary stone specimens (*n* = 44) were acquired during elective endoscopic procedures. Specimens were divided into subgroups by k-means cluster analysis depending on calcium and phosphorus concentrations. The concentration of urine OPN was determined and compared for each subgroup and the control group. **Results**: Cluster analysis divided the deposits into three clusters. Cluster 1 contained mainly calcium oxalate deposits; Cluster 2 contained uric acid deposits; Cluster 3 contained deposits with a high content of calcium phosphate. Urine OPN concentration in CaP stone-formers (5.77 ng/mL) differed significantly from those of controls (17.05 ng/mL, *p* = 0.013) and CaOx stone-formers (15.31 ng/mL, *p* = 0.048). **Conclusions**: The concentration of urine OPN varies depending on the elemental composition of renal calculi. The lowest concentration of OPN was determined in the group of patients with a high content of calcium phosphate in the deposits.

## 1. Introduction

The prevalence of urolithiasis varies between different populations. The average detection rate of urolithiasis in Western countries is approximately 10%. In some regions of North and East Asia, the rate ranges from 1% to 8%, and in Saudi Arabia, it can even reach up to 20% [1,2]. However, it should be stated that the above data refer to symptomatic urolithiasis as the detection of asymptomatic urolithiasis is difficult due to the lack of screening tests. Urolithiasis is a recurrent disease. A study of 7500 individuals from the German population found that over 22 years, 42% of patients had a relapse [3]. In the literature, the frequency of recurrence of urolithiasis is often generalized to 50% over a lifetime.

The crystal structure and elemental composition of urinary stones are variable—stones can have a homogeneous or heterogeneous composition. The most common substances forming kidney stones are outlined below:

Calcium Oxalate: The most common mineral found in deposits is whewellite (CaC_2_O_4_· H_2_O). Weddellite (CaC_2_O_4_·2H_2_O) is a less-stable crystalline form and transforms into whewellite over time [4]. Depending on the population studied, calcium oxalate is a component of about 75–90% of urinary stones. Stones composed exclusively of whewellite or weddellite account for about 36% of deposits [5,6].

Calcium Phosphate: Deposits composed only of hydroxyapatite are relatively rare (1–5%), although in combination with calcium oxalate they constitute a high percentage of kidney stones, ranging from 30% to 45% [6,7].

Uric Acid: Another component of urinary stones is uric acid (C_5_H_4_N_4_O_3_), which accounts for about 15% of the deposits, more often as the sole component [6,8].

Struvite: Struvite (NH_4_MgPO_4_·6H_2_O) is a component of about 8–15% of urinary stones, usually in deposits mixed with calcium oxalate or calcium phosphate [6]. Struvite stones are common in patients with recurrent urinary tract infections.

Proteins found in urine have an important impact on the formation of deposits, from matrix formation to inhibition or growth promotion. One of the key proteins with a broad effect on the process of urinary stone formation is osteopontin (OPN).

OPN is a phosphoprotein that influences bone mineralization and the attachment of osteoclasts to the bone surface; however, it is present in a wide variety of tissues. In the kidneys, it is found in the epithelium of the distal tubules and the collecting tubules of the nephrons [9]. OPN inhibits the nucleation and growth of deposits and hinders their attachment to the urothelium. On the other hand, it is an important component of the matrix of oxalate and calcium phosphate stones, and so its aggregation at the site of deposit formation can facilitate crystallization [10,11].

These contradictory properties of OPN lead to conflicting results in studies investigating the role of OPN influence in the development of renal calculi [12,13]. Studies assessing osteopontin concentrations in patients with nephrolithiasis have also yielded inconsistent results. Most available studies have been conducted on small patient groups, often without distinguishing the type of stones or focusing solely on calcium oxalate stones [14,15].

The presented study has two primary aims: Firstly, to determine the relationship between the concentration of urinary OPN in patients with nephrolithiasis and the type of their kidney stones. Additionally, we compared OPN levels in stone-formers with those in a group of control subjects with no history of urolithiasis.

## 2. Materials and Methods

Patients were qualified for lithotripsy procedures in the outpatient clinic or during previous hospitalizations on the basis of medical history, physical examination, computed tomography (CT), and ultrasound imaging. The urinary stones were obtained during elective endoscopic combined intrarenal surgery (ECIRS), percutaneous nephrolithotripsy (PCNL), or retrograde intrarenal surgery (RIRS) procedures performed at the I Urology Clinic of the Medical University of Łódź from 2023 to 2024.

All patients qualified for the procedure had a sterile urine culture. Patients presenting any signs of active urinary tract infection, both clinically and in urinalysis, were disqualified from the procedure. To avoid sample contamination and possible misrepresentation of urine parameters, patients with visible hematuria, pyuria, or turbid urine samples were disqualified from this study.

The control group consists of persons with no history of urolithiasis, no history of acute kidney injury, and no history of renal surgery. Absence of silent kidney stones was confirmed (in addition to lifelong negative urolithiasis history) if no kidney stones were detected in previous radiography, USG, or CT scans.

This study was approved by the University’s Ethics Committee, and all patients gave consent to be included in this study.

### 2.1. Urine Analysis

The urine samples were mid-stream urine, collected into sterile containers one day before the procedure and centrifuged (1600 rpm/min for 5 min). Five microliters of HALT protease inhibitor cocktail was added to each 0.5 mL sample and then frozen to −80 °C before transportation.

Urinary osteopontin (OPN) concentrations were determined using a commercially available Human Osteopontin ELISA kit (SEA899Hu, Cloud-Clone Corp., Katy, TX 77494 USA; lot numbers L230515661 and L230309604), according to the manufacturer’s instructions.

The assay range of the test was 0.625–40 ng/mL with a sensitivity of 0.237 ng/mL. Urine samples were diluted 5-fold prior to analysis. All samples and standards were measured in duplicate. Absorbance at 450 nm was measured using a VICTOR™ X4 Multilabel Plate Reader (PerkinElmer, Waltham, MA, USA). All washing steps were performed with a Stat-Matic Plate Washer II (Sigma-Aldrich, St. Louis, MO, USA).

Samples were stored at −80 °C and analyzed at the first thaw, without prior freeze–thaw cycles. All samples were centrifuged at 3000× *g* for 10 min, and the clarified supernatant was used for analysis. Obtained data were analyzed with WorkOut 2.5 software using a four-parameter logistic (4-PL) regression model for curve fitting. The concentration of the OPN in patient samples was quantified by interpolation from the corresponding standard curve. The intra-assay coefficient of variation (CV) was 2.34%, while the inter-assay CV was 9.68% for samples with an inhibitor and 10.72% for samples without an inhibitor.

### 2.2. Stone Specimen Analysis

Crystallography was performed via room temperature powder X-ray diffraction using a PANalytical X’Pert Pro MPD diffractometer (Malvern Panalytical Ltd., Royston, UK). The quantitative multi-elemental analysis was performed by Inductively Coupled Plasma–Optical Emission Spectroscopy (ICP-OES). The full description of steps and parameters of the stone specimen analysis is described in our previous study [16].

### 2.3. Statistical Analysis

The statistical analysis was carried out using RStudio, ver. 2025.05.0+496. K-means cluster analysis was performed using “cluster”, “plotly”, and “ggplot2” R Studio packages. The number of clusters for k-means cluster analysis was determined to be *n* = 3. Detailed algorithms used for assessment of number of clusters are provided in Supplementary Materials S1.

The normal distribution of the data was determined by the Shapiro–Wilk test.

The two-sided Student’s *t*-test was used to compare data with normal distribution—BMI, blood creatine, urinary OPN concentration—in the study and control groups.

Wilcoxon’s test *t* was used to compare data with non-normal distribution—calcium concentration, phosphorus concentration, OPN concentration—in clusters.

The study and control group characteristics are described using the medians with 95% confidence intervals for non-normal distribution and means with standard deviations for normal distribution.

## 3. Results

The clinical characteristics of the study group are presented in Table 1. The total number of participants was 44, including 20 men and 24 women. The average age in the study group was 63.1 years—63.5 years in the female group, and 62.7 years in the male group. The average BMI in the study group was 27.2 kg/m^2^; in the male group, the average BMI was 28.7 kg/m^2^, and in the female group, 26 kg/m^2^. The mean serum creatinine concentration was calculated separately for the groups of men and women (99.4 μmol/L and 75.3 μmol/L, respectively); the established normal range was 62–115 μmol/L for men and 53–97 μmol/L for women. Only three men and one woman presented creatinine levels above the normal range.

Crystallographic examination provided insight into the presence of individual minerals in urinary stone deposits. Homogeneous stones (*n* = 17, 38%) consist of struvite, uric acid, apatite, or whewellite. The most common crystal structure was weddellite—its presence was detected in 35 (79%) of the examined deposits. Apatite was present in twenty deposits (45%), uric acid in seven deposits (16%), and struvite was detected in the composition of three deposits (7%). Cystine, 2,8-dihydroxyadenine, drug-induced, or other rare stones were not detected.

The analysis of the deposits was performed based on the contents of calcium and phosphorus in stone specimens. The histograms and distribution of individual elements in the studied stones are presented as histograms in the Supplementary Materials (Supplementary Materials S2).

**Figure 1:**
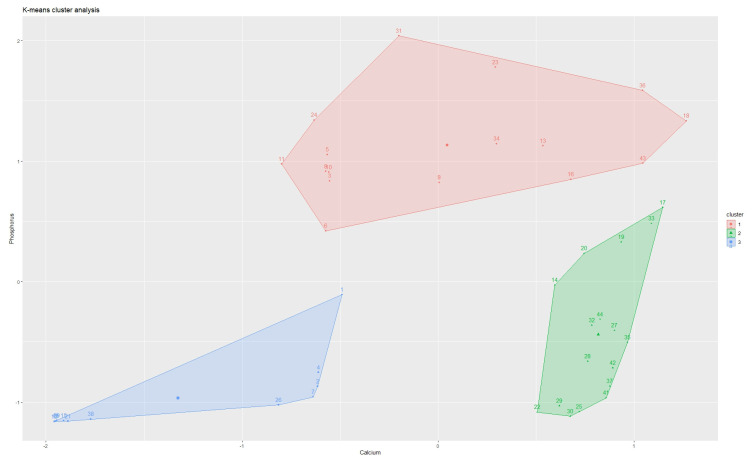
Results of cluster analysis and classification of deposits into subgroups: Cluster 1, *n* = 17; Cluster 2, *n* = 11; Cluster 3, *n* = 16.

The results of the k-means cluster analysis are presented in Figure 1. It is important to note that Figure 1 represents the same data points as Supplementary Materials S2 Figure S4, with the addition of a graphic representation of the cluster analysis.

The concentration of calcium and phosphorus was calculated for each individual cluster (Table 2). Notably, the results of the XRD analysis were not taken into account in the process of assigning stone specimens to designated clusters—the cluster analysis was based purely on the elemental composition of analyzed specimens to ensure the most objective results.

Comparison of calcium and phosphorus concentrations between clusters showed statistical significance (*p* < 0.01) in each pair for both elements. The cluster analysis had the intended effect: the deposits were differentiated into distinctive subgroups. For clarity, in the following text, Cluster 1 is described as CaOx; Cluster 2 is described as uric acid (UA); Cluster 3 is described as CaP. This choice is strongly dictated by the contents of calcium and phosphorus in individual clusters and highly significant (*p* < 0.01) disparities between clusters. Nevertheless, this is a form of simplification since kidney stones are heterogeneous and often consist of multiple components. Ultimately, the proposed classification of patients as CaP, UA, and CaOx stone-formers (instead of Cluster 1, Cluster 2, and Cluster 3 patients) is performed to simplify further discussion and better reflect clinical implications.

**Figure 2:**
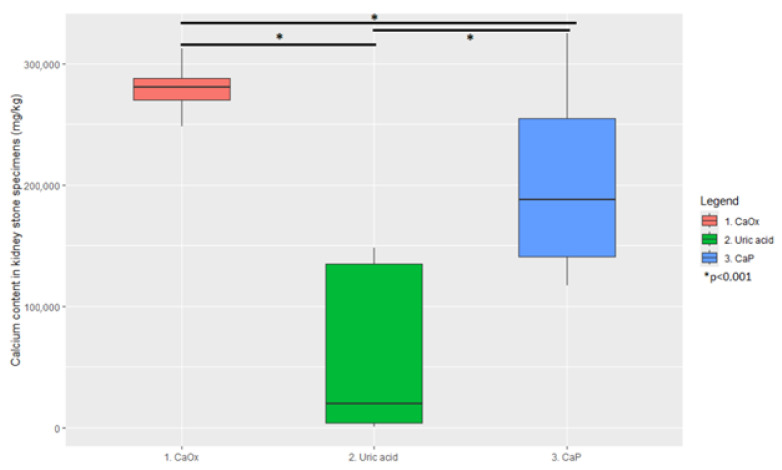
Comparison of stone specimens’ calcium content between clusters.

**Figure 3:**
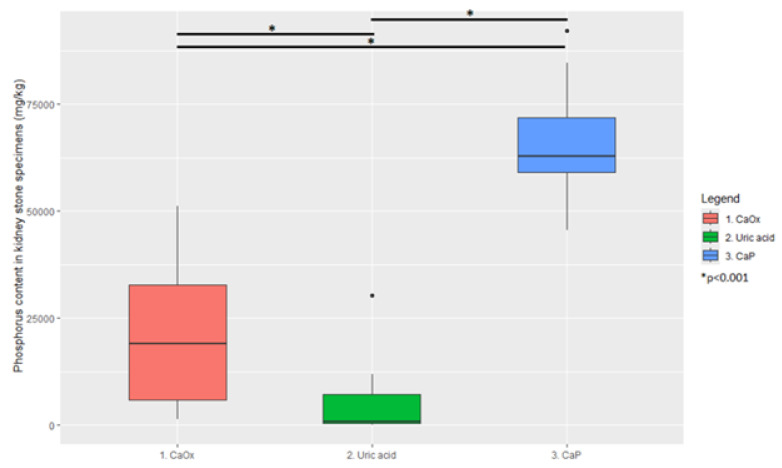
Comparison of stone specimens’ phosphorus content between clusters.

Graphical representations of the results are presented in Figure 2 and Figure 3.

Similar to other studies utilizing measurements of urine OPN measured by ELISA kits [17,18], the OPN levels are presented in ng/mL. The additional analyses—including the OPN normalization to urine creatinine (ng/mg Cr) and sensitivity analyses—are presented in Supplementary Materials S3.

**Figure 4:**
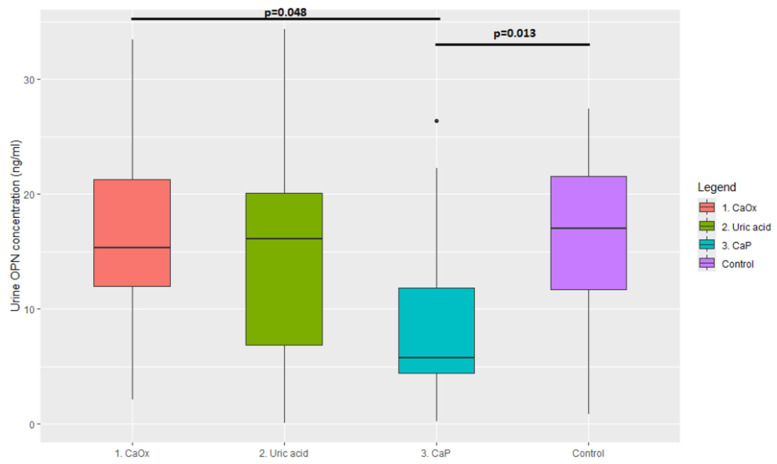
Comparison of urinary OPN concentration between stone-formers and control group.

The concentration of OPN was calculated for each cluster defined by the previous analysis, presented in Table 3 and Figure 4. Comparison of OPN concentrations between subgroups using Wilcoxon’s test showed a statistically significant difference in concentrations between Cluster 3 and the control group (*p* = 0.013) and between Cluster 3 and Cluster 1 (*p* = 0.048). The difference in OPN concentration between Cluster 3 and Cluster 2 was not statistically significant (*p* = 0.19). Comparison of the combined concentration of OPN in the study and control groups showed no statistically significant differences (*p* = 0.34).

## 4. Discussion

The study group consisted of 44 individuals, 24 (55%) women and 20 (45%) men. Despite advanced kidney stone disease, kidney function (measured by eGFR) in the vast majority of individuals remained within normal limits. This fact is highly relevant for osteopontin measurements as impaired kidney function can lead to elevated OPN secretion in both blood serum and urine [19,20]. It is possible that OPN concentration is characteristic of various kidney diseases, and the coexistence of chronic kidney disease with urolithiasis could result in false-positive findings. This should be taken into account when determining osteopontin concentration in the urine of individuals with kidney diseases overlapping with kidney stone disease.

In addition to phosphorus and calcium, the ICP-OES analysis included many other elements: Al, Ba, Cd, Co, Cr, Cu, Fe, K, Mg, Mn, Ni, Pb, S, Sr, Ti, and Zn. The values of these elements were not included in the final results of the analysis as many of them were present only in some of the deposits or did not have a direct impact on the assessment of the type of stone. The assessment of other elements, such as zinc or potassium, may provide valuable insight into metabolic processes involved in the formation and crystallization of renal calculi [21,22]. We have presented the results of trace elements analysis in renal calculi in a different paper [16].

In the studied group of 44 deposits, the distribution of uniform deposits was as follows: 15 deposits were composed exclusively of calcium oxalate (34%); 4 deposits of uric acid only (9%); 2 deposits of struvite only (4.5%); 2 deposits of calcium phosphate only (4.5%). The amount of mixed deposits is as follows: 16 (36.5%) were calcium oxalate deposits with calcium phosphate; 3 (7%) were calcium oxalate deposits with uric acid; 2 (4.5%) were calcium phosphate deposits with struvite. The occurrence of each type of deposit in the study group coincides with the frequency of deposits presented in the literature [23,24].

Kidney stone specimens were divided into three subgroups via k-means cluster analysis. Grouping deposits using this method, rather than arbitrary division, allowed for the most objective grouping of specimens. The presented results grade the subgroups according to the concentrations of both calcium (Cluster 1 > Cluster 3 > Cluster 2) and phosphorus (Cluster 3 > Cluster 1 > Cluster 2).

Group 1 included deposits with the highest concentration of calcium and, simultaneously, a low concentration of phosphorus. These deposits consist of either pure calcium oxalate or calcium oxalate with a small addition of calcium phosphate.

Group 2 included deposits that were practically devoid of calcium and phosphorus, mainly consisting of uric acid crystals. A crucial observation for such a group of deposits is that they crystallize most often due to pathomechanisms caused by other disease entities.

Group 3 contains deposits with the highest concentration of phosphorus, mainly in the form of apatite. Additionally, this group included two struvite deposits. Stone-formers included in this group may have a worse prognosis in the case of ESWL therapy, which is essential information for the choice of appropriate treatment [25].

The primary objective of this study was to investigate the variability in urine OPN concentration with the type of kidney stones. The presented results show a reduced concentration of OPN in patients with deposits rich in phosphorus (Cluster 3). The median concentration of OPN in Cluster 3 (5.77 ng/mL) was almost three times lower than in the other subgroups and the control group. The difference in OPN concentration between Clusters 1 and 3 (*p* = 0.048) is especially noteworthy as both subgroups include stones partially composed of calcium oxalate; they are differentiated by the higher calcium phosphate concentration in Cluster 3 specimens. In patients with no history of nephrolithiasis or other kidney diseases, urinary OPN concentrations were significantly higher than in Cluster 3 (*p* = 0.013) but comparable to other subgroups (Figure 4). The presented results indicate a relationship between the amount of calcium phosphate in kidney stones and the concentration of urine OPN. This observation may have diagnostic value in detecting apatite deposits through urinalysis, potentially establishing low urine OPN levels as a marker for apatite deposits.

This study has several limitations. Firstly, the study group is fairly small, including *n* = 44 patients—this results in quite modest clusters, as well as wide ranges of 95% CIs. Also, some results are on the border of statistical significance (especially the comparison between CaP and CaOx stone-formers, *p* = 0.048). This was due to strict exclusion and inclusion criteria: included patients had simultaneously advanced urolithiasis and normal kidney function; the urine specimens (and, consequently, stone specimens) were excluded in case of any visible impurities; and, on occasion, the amount of kidney stone specimen retrieved during the procedure was insufficient. Nevertheless, the results show clear differences both in the cluster analysis and in OPN levels between clusters.

Another limitation was accounting for possible confounders and differences between the control and study groups. Precise information on patients’ diet, hydration status, or lifestyle was often not reliable as most patients (especially the elderly) describe their habits as “normal”. A much more in-depth prospective cohort study on protein crystallization inhibitors accounting for such variables may provide valuable insight into the etiology of urolithiasis.

In the kidneys, OPN is physiologically involved in the mechanisms of immune response, especially in the process of recruitment and differentiation of M1 phenotype macrophages. Other influences of OPN include the recruitment of dendritic cells and activation of mastocytes. However, there are no clear connections between these findings and the formation of kidney stones [26].

Importantly, there is no clear consensus regarding the role of osteopontin in the pathogenesis of kidney stones; the papers and studies regarding this issue often present conflicting results. In a study by Chang et al., the impact of administering substances that promote kidney stone crystallization on mRNA expression of osteopontin (OPN) was assessed in a group of 60 mice. All tested compounds (ethylene glycol, vitamin D3, vitamin K, testosterone, and estradiol) led to a several-fold increase in OPN mRNA expression, indicating potential anti-crystallization properties [27]. These findings were confirmed by a study conducted by Wesson et al., in which mice with a genetic deletion of the osteopontin gene were compared to healthy controls. After four weeks of ethylene glycol administration, mice lacking the OPN gene developed significantly larger deposits than those in the control group. Moreover, ethylene glycol stimulation led to an increase in OPN levels in healthy mice [28].

An important aspect to consider is the mechanism by which osteopontin affects calcium phosphate, particularly hydroxyapatite. In an in vitro experiment, Li et al. investigated the influence of isolated protein domains of osteopontin on hydroxyapatite crystal formation. The study utilized a lipid membrane that mimicked the epithelial surface; the osteopontin domains demonstrated an inhibitory effect on both the adhesion of hydroxyapatite crystals to the lipid membrane and the nucleation process [29]. These findings were supported by a study by Wallace et al., which showed that osteopontin significantly inhibits the crystallization of calcium phosphate in artificial urine [30].

The propensity of OPN to bind and inhibit calcium phosphate crystallization is clearly reflected in the study results. Low OPN concentration was detected in patients suffering from kidney stones with high content of CaP. Future treatments may focus on increasing OPN levels in urine of CaP stone-formers, directly inhibiting crystallization of apatite crystals.

In vitro studies have demonstrated that OPN phosphorylation enhances the ability to inhibit the precipitation of deposits [15,31]. Experiments using electron microscopy imaging proved that OPN binds to the surface of hydroxyapatite crystals, prolongs the crystallization induction time, and inhibits the conversion of amorphous calcium phosphate into a crystal structure. The presence of OPN also caused a decrease in the size of hydroxyapatite crystallites [32,33]. An experiment by Stubbs et al. showed that OPN expression was significantly higher in the early stages of chronic kidney disease. Furthermore, in mice lacking the OPN gene (Spp1−/−), chronic kidney disease resulted in the deposition of significantly larger urinary stone deposits. During the same study, an in vitro experiment was also performed, stimulating the epithelial cells of the nephron tubules with calcium phosphate nanocrystals. It was observed that after only 72 h, the expression of the Spp1 gene was increased in cells stimulated with calcium phosphate compared to the control group [34].

Contrasting results were reported in a study by Okada et al., which also utilized mice with a double knockout of the osteopontin gene. Seven days after intraperitoneal administration of glyoxylic acid, the quantity and structure of the resulting CaOx crystals were assessed. The control group developed significantly larger CaOx crystals than the knockout group, and the CaOx crystals in the control group contained a matrix largely composed of OPN, suggesting a possible role of OPN in the formation of clinically relevant deposits [35].

One of the hypotheses explaining the reduced concentration of OPN in patients with calcium phosphate stones is the regulation of OPN secretion by calcitriol, the active form of vitamin D3. An increased amount of calcitriol stimulates the secretion of OPN both in bones and kidneys [36,37]. Calcitriol regulates calcium and phosphate metabolism by mobilizing phosphorus and calcium from bones in the case of hypophosphatemia [38]. Conversely, hyperphosphatemia is a factor that inhibits the production of calcitriol; low levels of calcitriol, in turn, result in low levels of OPN. Hyperphosphaturia facilitates the crystallization of apatite deposits while at the same time restraining calcitriol secretion, resulting in a decrease in OPN excretion in apatite stone-formers. It should be noted that the effects of phosphorus concentration in blood and urine on the secretion of the active form of vitamin D3 are not fully understood, and the presented hypothesis should be confirmed in future studies.

At the time of writing this paper, there are no approved therapeutic options targeting OPN production or excretion. The current research on OPN-targeting treatments is strictly experimental and performed only in vitro or on murine models. Development of genetic therapies or treatments targeting the OPN receptor may, in theory, modulate OPN expression, impacting urolithiasis and other kidney diseases [39].

## 5. Conclusions

The concentration of OPN in urine correlates with the elemental composition and crystal structure of kidney stones: CaP stone-formers had significantly lower urine OPN levels than CaOx stone-formers and the control group.

Our study indicates that patients with urinary osteopontin deficiency have a higher propensity for forming calcium-phosphate-rich stones. A deeper analysis, accounting for other predictive factors, may provide information regarding the value of urinary OPN as a marker for CaP stones.

## Figures and Tables

**Table 1:** Clinical characteristics of the study group and the control group.

	Study Group *n* = 44	Control Group *n* = 22	*p*-Value
Sex (female; male)	*n* = 24 (55%); *n* = 20 (45%)	*n* = 11 (50%); *n* = 11 (50%)	
Median age (years)	63.1	67.8	*p* = 0.1
BMI in male group (kg/m^2^)	28.7 ± 4.9	27.8 ± 2.76	*p* = 0.57
BMI in female group (kg/m^2^)	26 ± 4.6	24.7 ± 2.66	*p* = 0.31
Blood creatinine in male group (μmol/L)	99.4 ± 31.7	87.8 ± 20.1	*p* = 0.22
Average eGFR in male group (mL/min/1.73 m^2^)	74	83	
Blood creatinine concentration in female group (μmol/L)	75.3 ± 14.8	77.9 ± 28.3	*p* = 0.77
Average eGFR in female group (mL/min/1.73 m^2^)	76	71	
Urinary OPN concentration for both sexes (ng/mL)	13.43 ± 9.78	16.17 ± 5.87	*p* = 0.17
Urinary OPN concentration in men (ng/mL)	15.97 ± 9.2	17.51 ± 8.5	*p* = 0.72
Urinary OPN concentration in women (ng/mL)	11.23 ± 9.88	14.82 ± 7.23	*p* = 0.23
Longest stone dimension in CT (mean)	22.4 mm	-	
Longest stone dimension in CT (median)	21 mm	-	

BMI—body mass index; OPN—osteopontin; eGFR—estimated glomerular filtration rate; CT—computed tomography.

**Table 2:** Distribution of calcium and phosphorus concentrations in clusters.

Element	Cluster 1	Cluster 2	Cluster 3
Calcium (mg/kg)	280,600 (251,940–310,440 95% CI)	19,420 (868–145,275 95% CI)	187,800 (123,562–316,537 95% CI)
Phosphorus (mg/kg)	19,000 (1780–49,654 95% CI)	690 (137–25,732 95% CI)	62,725 (49,928–89,295 95% CI)

**Table 3:** Urinary concentration of OPN in patients from subgroups determined by cluster analysis.

Cluster 1, *n* = 17	Median = 15.31 ng/mL	95% CI = 2.18–32.39
Cluster 2, *n* = 11	Median = 16.12 ng/mL	95% CI = 0.52–32.23
Cluster 3, *n* = 16	Median = 5.77 ng/mL	95% CI = 0.62–24.92
Control group, *n* = 22	Median = 17.05 ng/mL	95% CI = 2.18–32.39

## Data Availability

All data, including ELISA test results, ICP-OES results, and XRD results, are available upon request.

## Supplementary Materials

The following supporting information can be downloaded at https://www.mdpi.com/article/10.3390/jcm14176247/s1, Supplementary Materials S1, S2, S3.
