# Supplementary material for: Correlation Between Urinary Osteopontin Concentration and the Mineral Content and Composition of Kidney Stones
Source: J Clin Med. 2025 Sep 4;14(17):6247. doi: 10.3390/jcm14176247 (PMC12429501; doi:10.3390/jcm14176247)
Supplement: Supplementary file 1 [file jcm-14-06247-s001.zip › jcm-3827819-supplementary.pdf]

Supplemet S1.

The amount of clusters were decided by a total of 29 different algorithms (R studio packages "easystats", "NbClust", "mclust"). The choice of 3 clusters is supported by 11 (37.93%) methods out of 29 (kl, Hartigan, Scott, Marriot, trcovw, Tracew, DB, Ratkowsky, Ball, PtBiserial, SDindex)

Figure S1. Algorithms indicating the optimal number of subgroups.

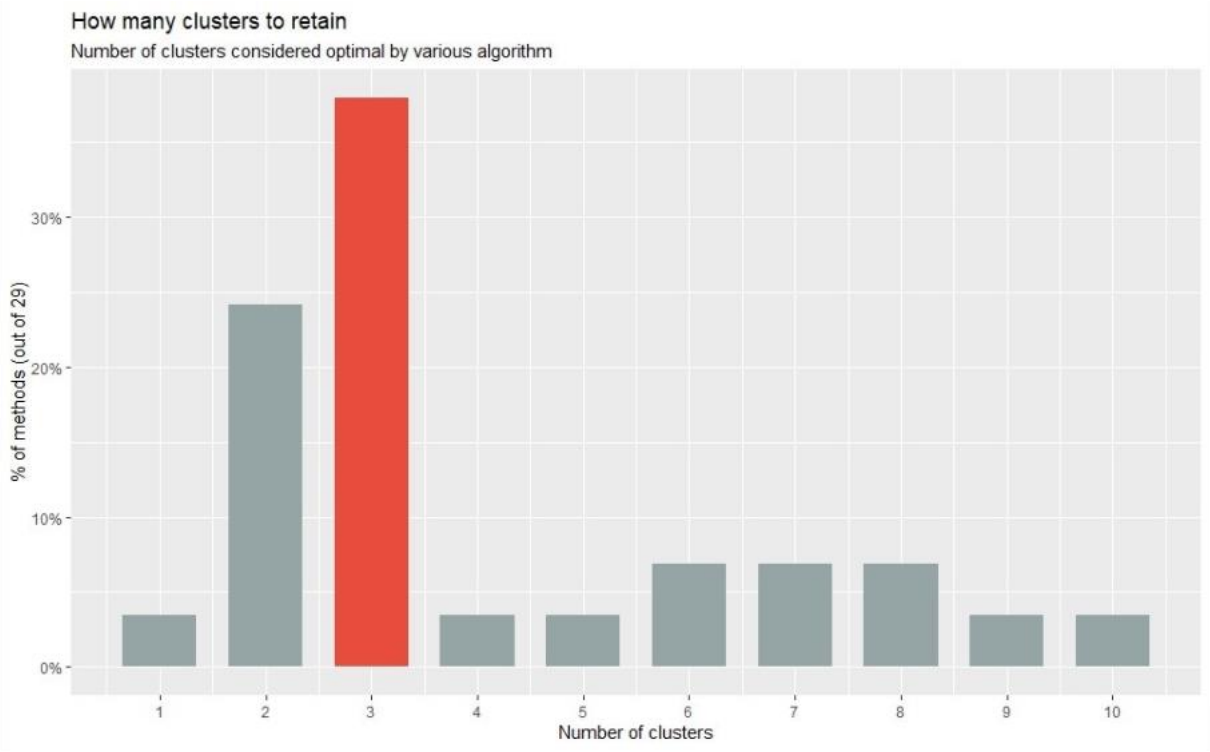

Supplement S2

Figure S2. Distribution of calcium concentration in the group of kidney stones, n=44

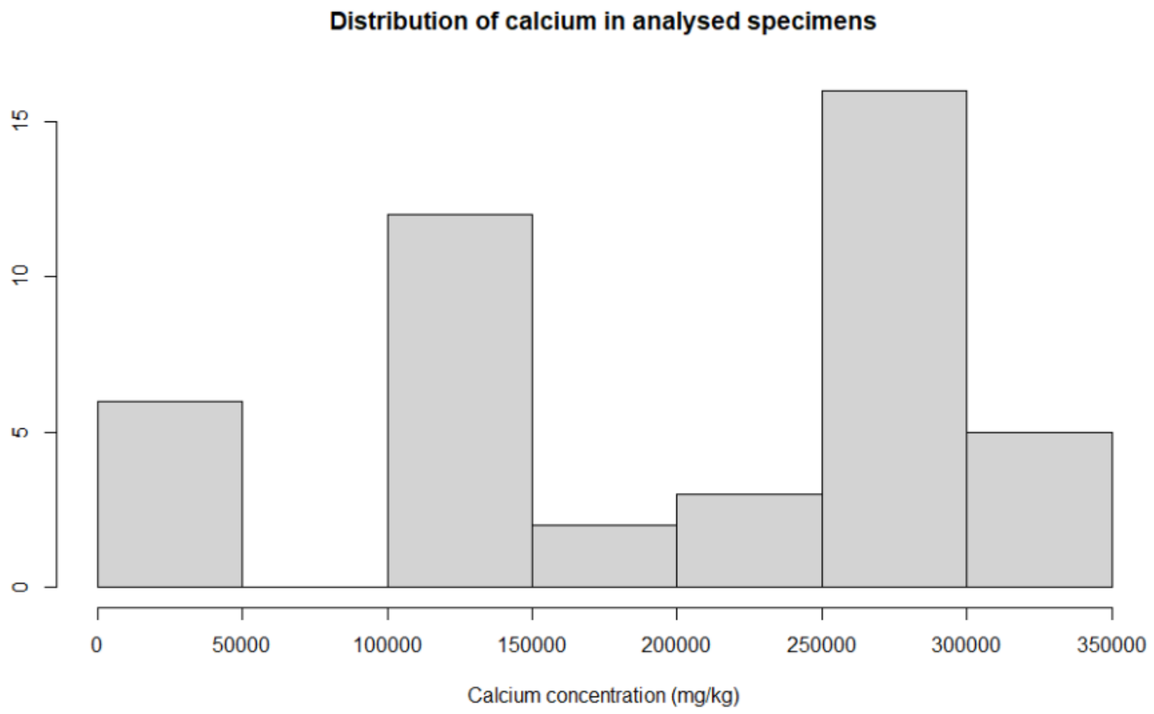

Figure S3. Distribution of phosphorus concentration in the group of kidney stones, n=44

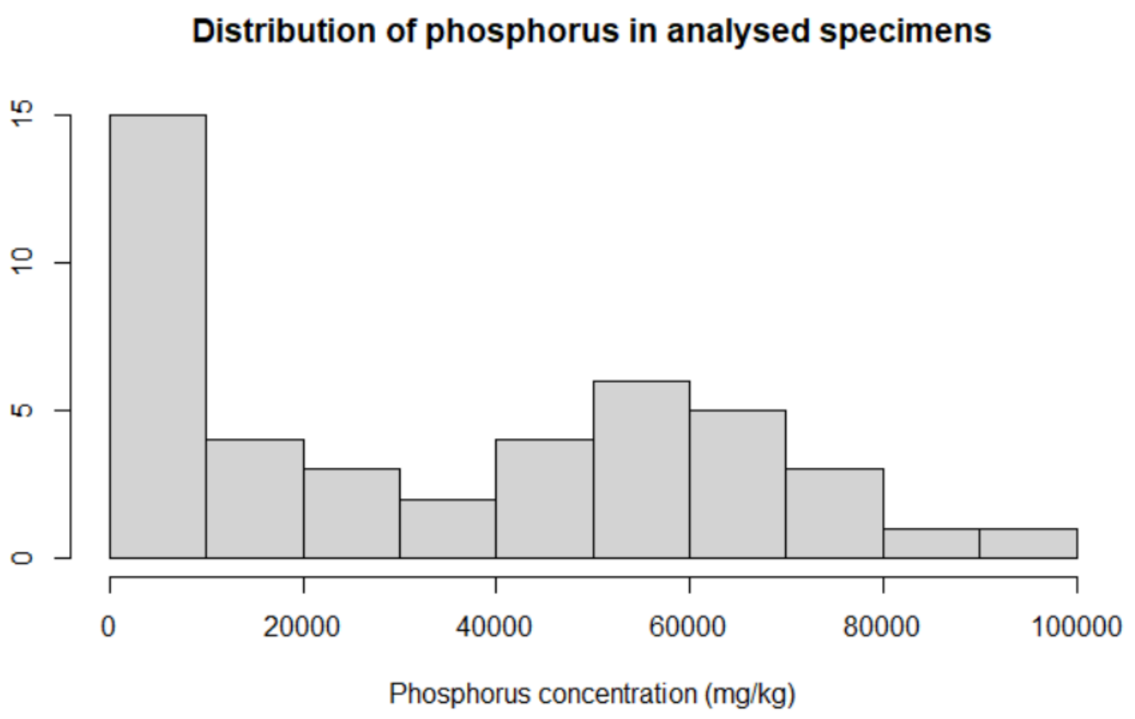

Figure S4. Calcium and potassium concentrations in urinary stone specimens, n=44. Each point of

the diagram corresponds to one specimen.

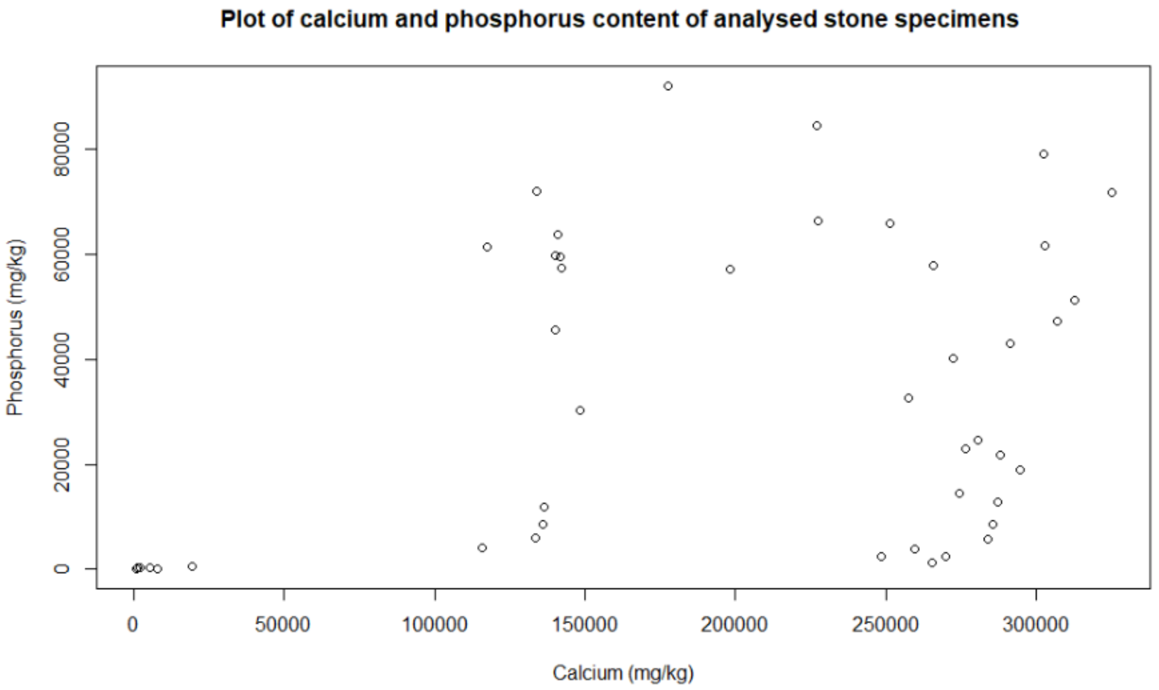

Supplement S3

The normalisation of OPN levels was performed by converting creatinine levels to mg/dL and than to mg/mL. The conversion factor was 88.42.
E.g. creatinine 100 µmol /L = 100/88.42 mg/dL = 1.13 mg/dL = 0.0113 mg/ml
Next, OPN levels (µg/ml) were divided by creatinine (mg/ml) resulting in OPN measured in µg/mg Cr units.

Table S1. Normalised urinary concentration of OPN in patients from subgroups determined by cluster analysis.

Cluster 1, n = 17	Median = **1511,78** µg/mg Cr	95% CI= 211.09-3111.55 µg/mg Cr
Cluster 2, n = 11	Median = **1438,30** µg/mg Cr	95% CI= 50.71 – 4206.81 µg/mg Cr
Cluster 3, n = 16	Median = **728,17** µg/mg Cr	95% CI= 69.78 – 2679.28 µg/mg Cr
Control group, n = 22	Median = **1833,24** µg/mg Cr	95% CI= 140.668 3681.705 µg/mg Cr

The results of Wilcoxon test for normalised OPN are p = 0.14, comparing Cluster 1 to Cluster 3, and p = 0.01, comparing Cluster 3 to Control group.

Secondly, we excluded the struvite stones from the analysed specimens to perform sensitivity analyses. Struvite stones commonly contain bacteria and may cause chronic infection, so inclusion of these stones may potentialy distort OPN excretion.

Table S2. Comparison of urinary OPN concentration in subgroups determined by cluster analysis with exclusion of struvite stone formers

Cluster 1, n = 17	Median = 15.31 ng/ml	95% CI= 2.18 – 32.39
Cluster 2, n = 11	Median = 16.12 ng/ml	95% CI= 0.52 – 32.23
Cluster 3, n = 14	Median = 5.77 ng/ml	95% CI= 0.62 – 24.92
Control group, n = 22	Median = 17.05 ng/ml	95% CI= 2.18 – 32.39

The results of Wilcoxon test for OPN levels in sensitivity analyses are p = 0.08, comparing Cluster 1 to Cluster 3, and p = 0.03, comparing Cluster 3 to Control group.

Considering, that the median and 95% CI are uniform with the results from analysis including struvite stones the inclusion of struvite stones is justified. The rise in p-values results from decrease in specimens included in Cluster 3.
